# Segmental and Multifocal Isolated Dystonias: Similarities and Differences

**DOI:** 10.1002/mdc3.70390

**Published:** 2025-10-11

**Authors:** Hyder A. Jinnah, Vittorio Velucci, Daniele Belvisi, Gamze Kilic‐Berkmen, Joel S. Perlmutter, Laura J. Wright, Christine Klein, Jeanne S. Feuerstein, Steven Bellows, Joseph Jankovic, Cynthia Comella, Richard L. Barbano, Aparna Wagle Shukla, Stephen G. Reich, Mark S. LeDoux, Alberto J. Espay, Kevin R. Duque, Florence C.F. Chang, Victor S.C. Fung, Sarah Pirio‐Richardson, Carmen Terranova, Emile S. Moukheiber, Sarah Idrissi, Barbara Vitucci, Susan H. Fox, Samuel Frank, Natividad Stover, Brian D. Berman, Rachel Saunders‐Pullman, William G. Ondo, Christopher L. Groth, Marcello Esposito, Laura Avanzino, Francesco Bono, Roberto Erro, Marcello Mario Mascia, Antonella Muroni, Alfredo Berardelli, Giovanni Defazio

**Affiliations:** ^1^ Department of Neurology Emory University School of Medicine Atlanta Georgia USA; ^2^ Department of Translational Biomedicine and Neuroscience University of Bari Aldo Moro Bari Italy; ^3^ Department of Human Neurosciences Sapienza University of Rome Rome Italy; ^4^ IRCCS Neuromed Institute Pozzilli Italy; ^5^ Department of Neurology, Radiology, Neuroscience, Physical Therapy and Occupational Therapy Washington University School of Medicine St. Louis Missouri USA; ^6^ Department of Neurology Washington University School of Medicine St. Louis Missouri USA; ^7^ Institute of Neurogenetics University of Luebeck Luebeck Germany; ^8^ Department of Neurology University of Colorado School of Medicine Aurora Colorado USA; ^9^ Department of Neurology, Rocky Mountain Regional VA Medical Center Aurora Colorado USA; ^10^ Parkinson's Disease Center and Movement Disorders Clinic, Department of Neurology Baylor College of Medicine Houston Texas USA; ^11^ Department of Neurological Sciences Rush University Medical Center Chicago Illinois USA; ^12^ Department of Neurology University of Rochester Rochester New York USA; ^13^ Department of Neurology, Norman Fixel Institute for Neurological Diseases University of Florida Gainesville Florida USA; ^14^ Department of Neurology University of Maryland School of Medicine Baltimore Maryland USA; ^15^ Veracity Neuroscience Memphis Tennessee USA; ^16^ James J. and Joan A. Gardner Family Center for Parkinson's Disease and Movement Disorders, Department of Neurology University of Cincinnati Cincinnati Ohio USA; ^17^ Movement Disorders Unit, Department of Neurology, Westmead Hospital Sydney New South Wales Australia; ^18^ Sydney Medical School University of Sydney Sydney New South Wales Australia; ^19^ Department of Neurology University of New Mexico Health Sciences Center Albuquerque New Mexico USA; ^20^ Department of Clinical and Experimental Medicine University of Messina Messina Italy; ^21^ Department of Neurology Johns Hopkins School of Medicine Baltimore Maryland USA; ^22^ Neurology Unit, “Dimiccoli” General Hospital Barletta Italy; ^23^ Division of Neurology University of Toronto Toronto Ontario Canada; ^24^ Beth Israel Deaconess Medical Center Harvard Medical School Boston Massachusetts USA; ^25^ Department of Neurology University of Alabama at Birmingham Birmingham Alabama USA; ^26^ VCU Parkinson's and Movement Disorders Center Virginia Commonwealth University School of Medicine Richmond Virginia USA; ^27^ Department of Neurology, Mount Sinai Beth Israel Medical Center New York New York USA; ^28^ Department of Neurology Icahn School of Medicine at Mount Sinai New York New York USA; ^29^ Department of Neurology Houston Methodist Neurological Institute Houston Texas USA; ^30^ Department of Neurology University of Iowa Iowa City Iowa USA; ^31^ Clinical Neurophysiology Unit, Antonio Cardarelli Hospital Naples Italy; ^32^ Department of Experimental Medicine, Section of Human Physiology University of Genoa Genoa Italy; ^33^ IRCCS Ospedale Policlinico San Martino Genoa Italy; ^34^ Center for Botulinum Toxin Therapy, Neurologic Unit Mater Domini University Hospital Catanzaro Italy; ^35^ Department of Medicine, Surgery and Dentistry “Scuola Medica Salernitana” University of Salerno Salerno Italy; ^36^ Neurology Unit University Hospital of Cagliari Cagliari Italy

**Keywords:** adult‐onset dystonia, idiopathic dystonia, isolated dystonia, multifocal dystonia, segmental dystonia

## Abstract

**Background:**

Whether the traditional distinction between segmental and multifocal dystonia is clinically or scientifically useful remains unclear.

**Objective:**

To evaluate whether idiopathic isolated adult‐onset segmental and multifocal dystonia can be differentiated based on clinical features other than the contiguity of affected body regions.

**Methods:**

We compared data on segmental and multifocal dystonia from two large dystonia databases established in the USA and Italy that used similar criteria for patient recruitment and assessment.

**Results:**

Compared to segmental dystonia, multifocal dystonia was characterized by a higher proportion of men, a younger age at dystonia onset, a greater frequency of upper limb dystonia, and a lower frequency of cranial dystonia at both onset and last examination. Segmental and multifocal dystonia had a similar frequency of alleviating maneuvers, non‐motor eye symptoms in blepharospasm, and neck pain and tremor in cervical dystonia. Although the initial spread pattern from focal to segmental or multifocal appeared faster in the segmental dystonia group, adjusting the analysis for the initial body site involved revealed no significant differences between the two groups. Segmental and multifocal dystonia starting in the same body site showed similar age, sex, and spread characteristics. The observed differences and similarities were consistent across both independent databases.

**Conclusions:**

Segmental and multifocal dystonia share differences and similarities. The observed differences may reflect a difference in the predominant site of dystonia onset. From a clinical perspective, therefore, the segmental/multifocal distinction is probably not valuable in the dystonia classification scheme, although further data may be needed from a pathophysiological perspective.

The distribution of affected body regions is a key criterion for classifying dystonia.^1^, ^2^ Dystonia is traditionally classified into focal (involving a single body region), segmental (affecting two or more contiguous regions), multifocal (involving two or more non‐contiguous regions), and generalized forms (affecting many body regions). Classification of dystonia by body distribution is relevant for both clinical and therapeutic management and is also useful for studies on pathophysiological mechanisms underlying the disease. It has been clearly demonstrated that the body distribution of dystonia may change with spread to other regions.^3^, ^4^ Focal dystonia may progress to segmental or multifocal forms, and segmental and multifocal types may generalize. In general, involvement of more body regions is considered to be more severe.

Whether the traditional distinction of segmental and multifocal dystonias is clinically or scientifically useful remains unclear.^2^ Many genetic and clinical studies examining the spread of dystonia do not differentiate between these two distributions, suggesting that the distinction may persist more from tradition than evidence. To date, no formal study has comprehensively evaluated whether segmental and multifocal dystonias in adults differ from each other clinically.

Two large databases on dystonia have been established in the USA and in Italy. The Dystonia Coalition (DC) database is a multicenter international effort addressing all types of isolated dystonia.^5^ The Italian Dystonia Registry (IDR) is a national database including patients with adult‐onset dystonia.^6^ Both databases use similar eligibility criteria for patient recruitment and assessment. In this study, we analyzed data from the DC and the IDR to investigate whether adult‐onset segmental and multifocal dystonia can be differentiated based on clinical features other than the contiguity of affected body regions.

## Methods

In February 2025, we screened the Dystonia Coalition (DC) and the Italian Dystonia Registry (IDR) databases for patients with isolated idiopathic adult‐onset dystonia (onset age ≥ 18 years).^1^ Since the main goal of this study was to determine whether there are meaningful differences between segmental and multifocal dystonias in adults, genetic etiologies were excluded as they are often generalized and typically present in childhood. The classification of dystonia as focal, segmental, multifocal, or generalized was based on the anatomical distribution of affected body regions, as defined by the Global Dystonia Rating Scale.^7^ According to this scale, the following regions were considered: upper face, lower face, neck, larynx, trunk, upper arm, hand, and lower limb.^8^, ^9^ Exclusion criteria included features suggesting acquired, genetic, or heredodegenerative dystonia, as well as exposure to neuroleptic drugs prior to dystonia onset.

Eligible patients were 2588 in the DC database (2250 with focal dystonia, 197 with segmental dystonia, 129 with multifocal dystonia, and 12 with generalized dystonia), 2052 in the IDR database (1611 with focal dystonia, 248 with segmental dystonia, 169 with multifocal dystonia, and 24 with generalized dystonia). The mean disease duration was 13.9 ± 10.7 years in the DC cohort and 13.6 ± 10.8 years in the IDR cohort.

The following demographic and clinical variables were available in both datasets: sex, age at data collection, age at dystonia onset, initial site of dystonia onset, age and year of dystonia localization in each body site. Additional dystonia‐related features assessed in both databases included the presence of alleviating maneuvers, non‐motor eye symptoms (such as burning sensation, grittiness, eye dryness, and photophobia)^10^ in patients with blepharospasm (BSP), and pain and head tremor in patients with cervical dystonia (CD).

Statistical analysis was conducted using STATA 11. Data were presented as mean ± standard deviation (SD) unless otherwise indicated. Normality of data was assessed with Q‐Q plots. Group differences were assessed using the *χ* ^2^ test or Fisher's exact test for categorical variables, and the Student's *t*‐test or the Mann–Whitney *U* test for continuous variables, as appropriate. Dystonia duration was measured from the year of dystonia onset to the year of the last examination. The spread of dystonia was estimated using Kaplan–Meier curves (compared by log‐rank test) and Cox regression analysis, with study time defined as the interval between focal dystonia onset and involvement of a second body region. Significance was set at the 0.05 level.

## Results

At the time of analysis, a total of 326 DC patients and 417 IDR patients had segmental or multifocal dystonia. The two series did not significantly differ in sex distribution (230 women and 96 men in the DC series vs. 284 women and 133 men in the IDR series, *P* = 0.5). The DC series was characterized by lower age at study (63.0 ± 10.8 years vs. 69.3 ± 11.1 years, *P* < 0.0001) and lower age at dystonia onset (49.2 ± 12.9 vs. 55.6 ± 13.6 years, *P* < 0.0001), whereas duration of disease was similar in both series (13.9 ± 10.7 vs. 13.7 ± 10.8 years, *P* = 0.8).

### Segmental versus Multifocal Dystonia: Demographic and Clinical Features

#### Dystonia Coalition (DC) Database

Compared to patients with segmental dystonia, those with multifocal dystonia showed a higher proportion of males (with both showing female predominance), lower age at dystonia onset, longer disease duration, lower frequency of cranial involvement, and a higher frequency of upper limb involvement (Table 1). Segmental and multifocal groups did not significantly differ for frequency of alleviating maneuvers, non‐motor eye symptoms in BSP, neck pain and neck tremor in CD (Table 1).

**TABLE 1:** Demographic and clinical features of segmental and multifocal dystonia in the Dystonia Coalition (DC) cohort

	Segmental dystonia	Multifocal dystonia	*P*
Sample size, n	197	129	–
Sex, men/women	48/149	48/81	0.013
Age, mean years ± SD	63.8 ± 11.4	62.1 ± 11.9	0.2
Disease duration, mean years ± SD	11.8 ± 9.3	17.1 ± 11.9	< 0.0001
Age at dystonia onset, mean years ± SD	51.8 ± 12.4	45.5 ± 12.9	< 0.0001
Distribution of dystonia at last examination			
Blepharospasm, n (%)	116 (58.9)	55 (42.6)	0.004
Oromandibular dystonia, n (%)	150 (76.1)	15 (11.6)	< 0.0001
Laryngeal dystonia, n (%)	34 (17.3)	29 (22.5)	0.2
Cervical dystonia, n (%)	120 (60.9)	114 (88.4)	< 0.0001
Task‐specific upper limb dystonia, n (%)	20 (10.2)	43 (33.3)	< 0.0001
Non‐task‐specific upper limb dystonia, n (%)	22 (11.2)	46 (35.7)	< 0.0001
Lower limb dystonia, n (%)	2 (1.0)	15 (11.6)	< 0.0001
Truncal dystonia, n (%)	8 (4.1)	0 (0.0)	0.024
Presence of alleviating maneuvers, n (%)	114/197 (57.9)	71/129 (55.0)	0.6
Non‐motor eye symptoms in blepharospasm, n (%)	55/116 (47.4)	26/55 (47.3)	0.99
Neck pain in cervical dystonia, n (%)	57/120 (47.5)	55/114 (48.2)	0.9
Head tremor in cervical dystonia, n (%)	70/120 (58.3)	64/114 (56.1)	0.7
Focal vs. segmental/multifocal onset, n (%)	95 (48.2) vs. 102 (51.8)	70 (54.3) vs. 59 (45.7)	0.3
Body distribution of dystonia at onset in patients with focal onset			
Blepharospasm, n (%)	53/95 (55.8)	20/70 (28.6)	0.0005
Oromandibular dystonia, n (%)	11/95 (11.6)	1/70 (1.4)	0.014
Laryngeal dystonia, n (%)	3/95 (3.2)	3/70 (4.3)	0.7
Cervical dystonia, n (%)	26/95 (27.4)	28/70 (40.0)	0.1
Task‐specific upper limb dystonia, n (%)	0/95 (0.0)	10/70 (14.3)	0.0001
Non‐task‐specific upper limb dystonia, n (%)	0/95 (0.0)	8/70 (11.4)	0.0008
Lower limb dystonia, n (%)	0/95 (0.0)	0/70 (0.0)	1.0
Truncal dystonia, n (%)	2/95 (2.1)	0/70 (0.0)	0.5
Body distribution of dystonia at onset in patients with segmental/multifocal onset			
Blepharospasm, n (%)	55/102 (53.9)	26/59 (44.1)	0.2
Oromandibular dystonia, n (%)	73/102 (71.6)	6/59 (10.2)	< 0.0001
Laryngeal dystonia, n (%)	14/102 (13.7)	8/59 (13.6)	0.98
Cervical dystonia, n (%)	61/102 (59.8)	53/59 (89.8)	< 0.0001
Task‐specific upper limb dystonia, n (%)	19/102 (18.6)	39/59 (66.1)	< 0.0001
Non‐task‐specific upper limb dystonia, n (%)	2/102 (2.0)	16/59 (27.1)	< 0.0001
Lower limb dystonia, n (%)	17/102 (16.7)	23/59 (39.0)	0.002
Truncal dystonia, n (%)	1/102 (1.0)	7/59 (11.9)	0.004

Abbreviation: SD, standard deviation.

Among patients with segmental dystonia, 95 individuals (48.2%) reported that dystonia began in a single body region (ie, had focal onset); similarly, 70 patients (54.3%) in the multifocal group reported a focal onset (*P* = 0.3). The remaining patients in both groups reported a near‐simultaneous onset of dystonia in two contiguous or non‐contiguous regions, precluding a clear classification of the initial presentation (Table 1). Regardless of the reported mode of onset, multifocal dystonia most commonly began in the upper limbs and the neck and less frequently in the cranial muscles, whereas segmental dystonia most commonly began in the face (Table 1).

**Figure 1:**
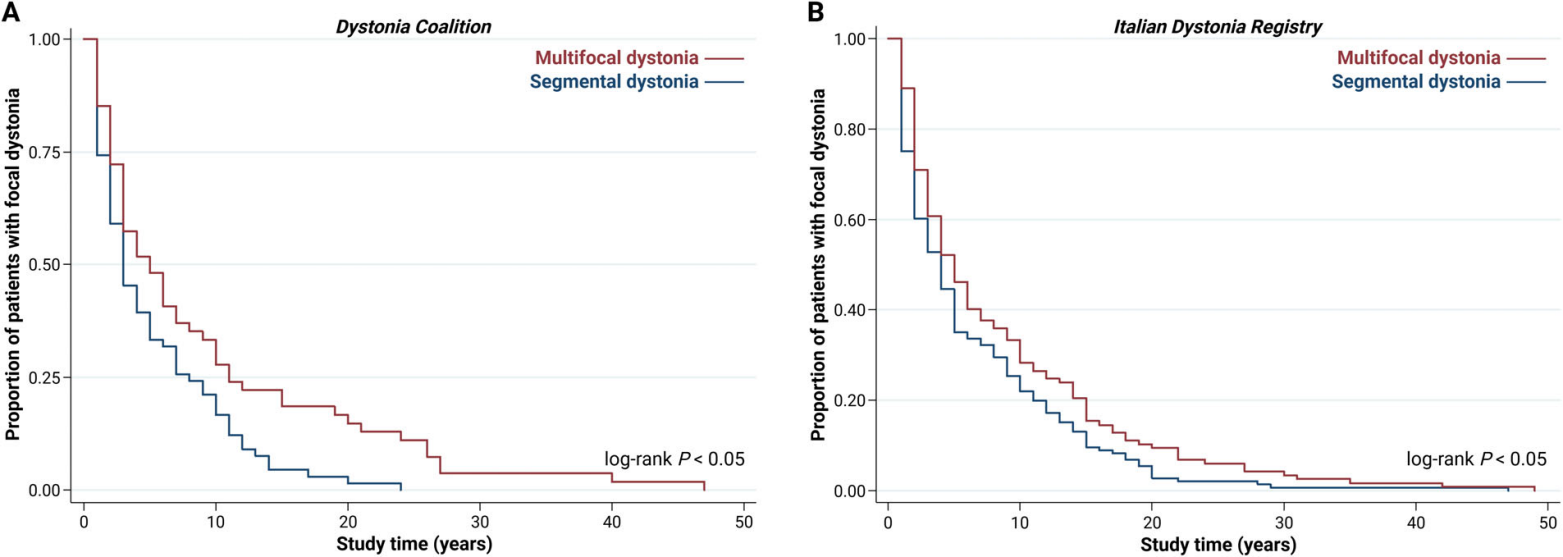
Kaplan–Meier survival analysis of dystonia spread in 120 patients from the Dystonia Coalition (A) and in 266 patients from the Italian Dystonia Registry (B) who initially presented with focal dystonia and later developed segmental or multifocal dystonia.

To assess the dystonia spread leading to segmental or multifocal forms, we focused on the 165 patients who reported focal dystonia onset. Since data on the year of dystonia onset and/or the year of first dystonia spread were not available in 45 patients, survival analysis was performed in 120 patients. Kaplan–Meier survival curves indicated a faster rate of spread in the multifocal group (Fig. 1A). However, Cox analysis adjusted by the first body localization of dystonia (cranial vs. cervical vs. upper limb vs. other sites) yielded no significant difference in spread speed between segmental and multifocal dystonia (Table 2).

**TABLE 2:** Cox survival analysis of spread of dystonia in 120 patients from the Dystonia Coalition (DC) and 266 patients from the Italian Dystonia Registry (IDR) who reported focal onset and later developed segmental or multifocal dystonia

Adjustment	Dystonia Coalition (DC)	Italian Dystonia Registry (IDR)
	Hazard ratio (95% CI), *P*	Hazard ratio (95% CI), *P*
None	0.62 (0.42 to 0.91), 0.015	0.78 (0.61 to 0.99), 0.044
Age	0.57 (0.39 to 0.85), 0.005	0.69 (0.53 to 0.89), 0.004
Sex	0.58 (0.39 to 0.87), 0.006	0.70 (0.54 to 0.91), 0.007
Type of dystonia at onset	0.73 (0.48 to 1.10), 0.1	0.81 (0.62 to 1.10), 0.1

*Note*: Hazard ratios refer to the rate of spread from focal to multifocal dystonia compared to the rate of spread from focal to segmental dystonia (reference group), adjusting for relevant variables.

Abbreviation: CI, confidence interval.

#### Italian Dystonia Registry (IDR) Database

Data from the IDR were largely similar to those observed in the DC cohort. Compared to patients with segmental dystonia, those with multifocal dystonia showed a higher proportion of males, similar disease duration, lower frequency of oromandibular dystonia, higher frequency of upper limb involvement, and a lower age at dystonia onset (Table 3). Segmental and multifocal groups did not significantly differ in frequency of alleviating maneuvers, non‐motor eye symptoms in blepharospasm, neck pain and neck tremor in cervical dystonia (Table 3).

**TABLE 3:** Demographic and clinical features of segmental and multifocal dystonia in the Italian Dystonia Registry (IDR) cohort

	Segmental dystonia	Multifocal dystonia	*P*
Sample size, n	248	169	–
Sex, men/women	68/180	65/104	0.018
Age, mean years ± SD	70.2 ± 11.3	67.3 ± 13.2	0.001
Disease duration, mean years ± SD	13.5 ± 10.3	14.1 ± 11.4	0.3
Age at dystonia onset, mean years ± SD	57.2 ± 12.4	53.2 ± 15.0	0.003
Distribution of dystonia at last examination			
Blepharospasm, n (%)	166 (66.9)	102 (60.4)	0.2
Oromandibular dystonia, n (%)	198 (79.8)	21 (12.4)	< 0.0001
Laryngeal dystonia, n (%)	58 (23.4)	22 (13.0)	0.008
Cervical dystonia, n (%)	121 (48.8)	128 (75.7)	< 0.0001
Task‐specific upper limb dystonia, n (%)	1 (0.4)	40 (23.7)	< 0.0001
Non‐task‐specific upper limb dystonia, n (%)	9 (3.6)	54 (32.0)	< 0.0001
Lower limb dystonia, n (%)	1 (0.4)	21 (12.4)	< 0.0001
Truncal dystonia, n (%)	7 (2.8)	2 (1.2)	0.3
Presence of alleviating maneuvers, n (%)	80/248 (32.3)	61/169 (36.1)	0.4
Non‐motor eye symptoms in blepharospasm, n (%)	92/166 (55.4)	49/102 (48.0)	0.2
Neck pain in cervical dystonia, n (%)	68/121 (56.2)	67/128 (52.3)	0.5
Head tremor in cervical dystonia, n (%)	68/121 (56.2)	66/128 (51.6)	0.5
Focal vs. segmental/multifocal onset, n (%)	149 (60.1) vs. 99 (39.9)	117 (69.2) vs. 52 (30.8)	0.1
Body distribution of dystonia at onset in patients with focal onset			
Blepharospasm, n (%)	95/149 (63.8)	55/117 (47.0)	0.006
Oromandibular dystonia, n (%)	8/149 (5.4)	3/117 (2.6)	0.4
Laryngeal dystonia, n (%)	10/149 (6.7)	1/117 (0.9)	0.026
Cervical dystonia, n (%)	34/149 (22.8)	33/117 (28.2)	0.3
Task‐specific upper limb dystonia, n (%)	0/149 (0.0)	14/117 (12.0)	< 0.0001
Non‐task‐specific upper limb dystonia, n (%)	2/149 (1.3)	10/117 (8.5)	0.006
Lower limb dystonia, n (%)	0/149 (0.0)	2/117 (1.7)	0.2
Truncal dystonia, n (%)	0/149 (0.0)	0/117 (0.0)	1.0
Body distribution of dystonia at onset in patients with segmental/multifocal onset			
Blepharospasm, n (%)	65/99 (65.7)	21/52 (40.4)	0.003
Oromandibular dystonia, n (%)	81/99 (81.8)	5/52 (9.6)	< 0.0001
Laryngeal dystonia, n (%)	19/99 (19.2)	6/52 (11.5)	0.2
Cervical dystonia, n (%)	42/99 (42.4)	38/52 (73.1)	0.0004
Task‐specific upper limb dystonia, n (%)	1/99 (1.0)	13/52 (25.0)	< 0.0001
Non‐task‐specific upper limb dystonia, n (%)	3/99 (3.0)	21/52 (40.4)	< 0.0001
Lower limb dystonia, n (%)	0/99 (0.0)	4/52 (7.7)	0.013
Truncal dystonia, n (%)	4/99 (4.0)	0/52 (0.0)	0.3

Abbreviation: SD, standard deviation.

Dystonia had a focal onset in 149/248 patients (60.1%) of the segmental dystonia group and in 117/169 patients (69.2%) of the multifocal group (*P* = 0.1). The remaining patients in both groups reported a near‐simultaneous onset in two contiguous or non‐contiguous regions, making it difficult to determine if there was a clear focal onset (Table 3). Regardless of the mode of onset, multifocal dystonia more frequently began in the upper limb and neck regions, and less frequently in the cranial region (Table 3).

In the 266 patients with focal dystonia onset, Kaplan–Meier survival curves suggested a faster spread in the multifocal group (Fig. 1B) than the segmental group. However, the difference in the pattern of spread between segmental and multifocal dystonia lacked significance on Cox analysis adjusted for the first body region affected by dystonia (Table 2).

### Segmental versus Multifocal Dystonia Starting in the Same Body Site

To see whether differences between segmental and multifocal dystonia were also present when these two forms of dystonia had a similar body site at onset, we compared segmental and multifocal types of dystonia starting in the same site. This analysis was limited to segmental and multifocal dystonia starting with BSP because only these two groups had a sufficient number of cases for statistical comparison. Segmental and multifocal patients from both databases who started as BSP at onset did not significantly differ for sex distribution, age at dystonia onset and pattern of spread (Table 4).

**TABLE 4:** Comparison of sex distribution, age at dystonia onset, and rate of spread in patients from the Dystonia Coalition (DC) and the Italian Dystonia Registry (IDR) who presented with focal blepharospasm at onset and later developed segmental or multifocal dystonia

	Dystonia Coalition (DC)			Italian Dystonia Registry (IDR)		
	Segmental dystonia at last examination	Multifocal dystonia at last examination	*P*	Segmental dystonia at last examination	Multifocal dystonia at last examination	*P*
Sex, men/women	15/38	9/11	0.2	28/67	16/39	0.9
Age at dystonia onset, mean years ± SD	53.2 ± 9.5	56.1 ± 5.6	0.1	58.7 ± 10.8	56.2 ± 12.8	0.1
Spread from focal blepharospasm to multifocal versus segmental dystonia, hazard ratio (95% CI)^a^	0.83 (0.48–1.47)		0.5	0.90 (0.65–1.26)		0.5

^a^ Hazard ratios refer to the rate of spread from focal blepharospasm to multifocal dystonia compared to the rate of spread from focal blepharospasm to segmental dystonia (reference group).

Abbreviations: CI, confidence interval; SD, standard deviation.

## Discussion

**Figure 2:**
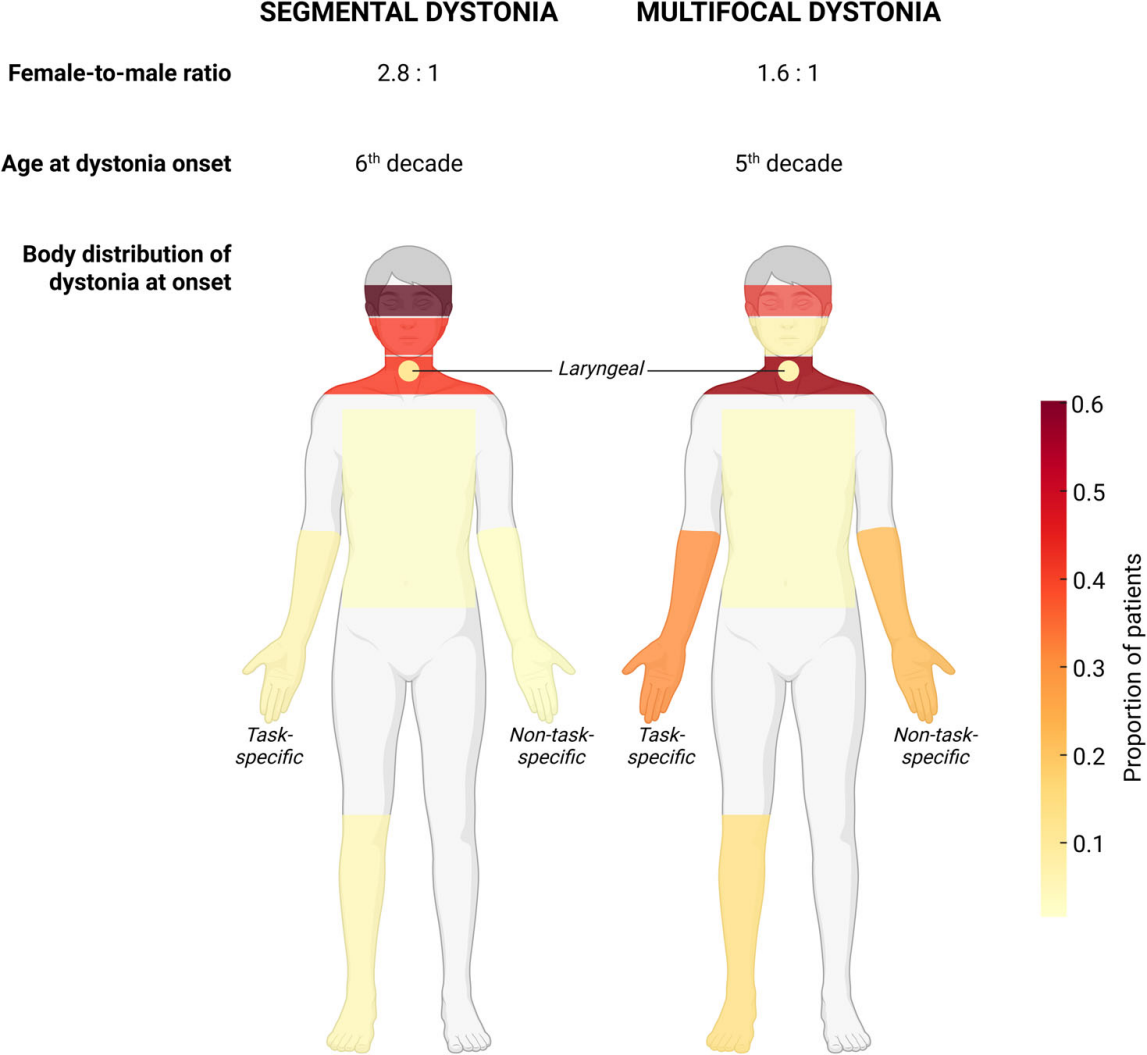
Relevant clinical differences between segmental and multifocal dystonia at disease onset.

Data from both the DC and IDR databases showed that segmental and multifocal dystonia differed in several features but also shared some similarities. Compared to segmental dystonia, multifocal dystonia was characterized by a higher proportion of men, a younger age at dystonia onset, a greater frequency of upper limb dystonia and a lower frequency of cranial dystonia at both onset and last examination, as illustrated in Figure 2. Despite these differences, segmental and multifocal dystonias had a similar frequency of alleviating maneuvers, non‐motor eye symptoms in BSP, and neck pain and neck tremor in CD. Although the initial spread pattern from focal to segmental or multifocal appeared faster in the segmental dystonia group, adjusting the analysis for the initial body site involved revealed no significant differences between the two groups.

The observation that differences and similarities between segmental and multifocal dystonias were comparable in the two independent cohorts of patients affected by dystonia helps validate the results obtained in the present study. Since dystonia starting in the upper limb predominated in the multifocal group, whereas cranial dystonia predominated in the segmental group, the different age‐ and sex‐related features of upper limb dystonia and cranial dystonia might explain the corresponding differences in age at dystonia onset and sex distribution between multifocal and segmental dystonia groups.^11^ The lack of substantial clinical differences between segmental and multifocal dystonia was further supported by two observations: (i) spread of dystonia was apparently slower in the multifocal group but the difference lacked significance after adjusting for dystonia localization at onset; and (ii) comparison of patients with segmental and multifocal dystonias starting in the same body part (ie, BSP, the largest sized subgroup with dystonia starting in the same body site) showed that patients who manifested focal BSP at onset and later developed segmental or multifocal dystonia did not differ in sex distribution, age at dystonia onset and pattern of spread.

Our study has some limitations. This was not a population‐based study. However, the recruitment criteria yielded case series that closely resemble the general population of adult‐onset dystonia in terms of both demographic and clinical characteristics.^5^, ^6^ Additionally, the consistency of findings across both independent databases strengthens the validity of our results. The difference in the age at dystonia onset between DC and IDR populations likely stemmed from differences in the source populations of the two registries. Nevertheless, it is unlikely that the slightly longer disease duration in the multifocal group from the DC database influenced comparisons, as adult‐onset segmental and multifocal dystonias rarely progress to generalized forms. The variable frequency of cranial and upper limb dystonia in segmental and multifocal groups might be due to the difficulty in examining body regions between the neck and hand; it is known that cranial and hand dystonia are more easily diagnosed in comparison to shoulder dystonia. Among dystonia‐associated features, we did not consider family history of dystonia because information on this topic was not ascertained by clinical examination of at‐risk relatives in both databases and therefore is unlikely to be reliable.^12^

Despite the foregoing limitations, our analysis provided novel information highlighting the variability in several phenotypic features between segmental and multifocal dystonias. There was a higher proportion of men, younger age at onset, and greater upper limb involvement in multifocal dystonia and a higher proportion of women, older age at onset, and greater cranial involvement in segmental dystonia. However, these differences may reflect only differences in the predominant site of dystonia onset. In line with this view was the observation that segmental and multifocal dystonias starting in the same body site showed similar age, sex, and spread features. It remains to be understood whether variable pathophysiological mechanisms may underlie the evolution of a focal dystonia toward segmental or multifocal trajectories. Indeed, age‐related differences of sensorimotor cortex plasticity in adults extend beyond the affected body part in focal dystonia, thus raising the possibility that age at onset is associated with different expression of dystonia spread.^13^, ^14^ Alternatively, the development of segmental or multifocal dystonia starting from the same initial dystonic localization may result from the encounter with specific risk factors over time, such as occupational exposures.^15^, ^16^

Given the current state of knowledge, there is limited evidence to support maintaining the distinction between segmental and multifocal dystonias in the dystonia classification scheme. However, even though the segmental/multifocal distinction is probably not valuable in idiopathic adult‐onset dystonia from a clinical perspective (because of the relatively minor differences between the groups), we may need more data from a scientific perspective to more definitively determine its value. Importantly, our findings should be interpreted in the context of the study population, which excluded childhood‐ and adolescent‐onset cases as well as genetic and acquired forms of dystonia. Therefore, we recommend temporarily maintaining the distinction in order to encourage further investigations, including studies addressing questions emerging from our analysis, such as the evolution of focal dystonia in a specific body site toward segmental or multifocal trajectories.

## Author Roles

(1) Research project: A. Conception, B. Organization, C. Execution; (2) Statistical Analysis: A. Design, B. Execution, C. Review and Critique; (3) Manuscript: A. Writing of the first draft, B. Review and Critique.

H.A.J.: 1A, 1B, 1C, 2A, 2B, 3A.

V.V.: 1C, 2C, 3A.

D.B.: 1C, 2C, 3A.

G.K.B.: 1C, 2C, 3A.

A.B.: 1C, 2C, 3A.

G.D.: 1A, 1B, 1C, 2A, 2B, 3A.

All the other authors: 1C, 3B.

## Disclosures

**Ethical Compliance Statement:** For the analysis of de‐identified data from the Dystonia Coalition, approval of the Emory University Human Subjects Review Board was obtained. For the Italian Dystonia Registry, the study was approved by the local ethics committee of the IRCCS Oncologico of Bari (Protocol No. 596, October 24, 2023). For the present study, the requirement for informed consent was waived because the study was register‐based, and individuals were not identifiable at any time. We confirm that we have read the Journal's position on issues involved in ethical publication and affirm that this work is consistent with those guidelines.

**Funding Sources and Conflicts of Interest:** No specific funding was received for this work. The authors declare that there are no conflicts of interest relevant to this work.

**Financial Disclosures for the Previous 12 Months:** J.S.P. has received funding from the Dystonia Coalition, the Barnes‐Jewish Hospital Foundation, and the Murphy Fund. L.J.W. has received funding from the Dystonia Coalition. M.S.L.D. is a speaker for Teva and Amneal. His clinical and laboratory research have been funded by the National Institutes of Health, Department of Defense, Revance, Cerevel, Cerevance, Annovis, Eon, Sage Therapeutics, Inhibikase Therapeutics, UCB Pharma, Intra‐Cellular Therapies, Ventus Therapeutics, the Michael J. Fox Foundation, the Dystonia Medical Research Foundation, and the Benign Essential Tremor Research Foundation. A.J.E. has received grant support from the NIH and the Michael J. Fox Foundation. He has received personal compensation as a consultant or scientific advisory board member for Mitsubishi Tanabe Pharma America, Amneal, Acorda, Abbvie, Bial, Supernus, NeuroDiagnostics Inc. (SYNAPS Dx), Intrance Medical Systems Inc., Merz, Praxis Precision Medicines, Citrus Health, and Herantis Pharma. He has also received publishing royalties from Lippincott Williams & Wilkins, Cambridge University Press, and Springer. S.H.F. has received clinical support from the Edmond J. Safra Foundation for Parkinson Research, the Parkinson Foundation, and the UHN Foundation. She has received research funding from the Michael J. Fox Foundation for Parkinson Research, NIH (Dystonia Coalition), Parkinson Canada, and the Weston Foundation. She has received honoraria from the International Parkinson and Movement Disorder Society, consultancy/speaker fees from Abbvie, Lundbeck, and Sunovion, and royalties from Oxford University Press. She also served as site PI for clinical trials for Biotie.

B.D.B. has received research support from the Dystonia Coalition (supported by NIH grant U54 NS116025), the Benign Essential Blepharospasm Research Foundation, the Parkinson's Foundation, VCU School of Medicine, Administration for Community Living, Dystonia Medical Research Foundation, and Neurocrine Biosciences. He has also received honoraria from the International Parkinson and Movement Disorder Society and served as a consultant for the Dystonia Medical Research Foundation. W.G.O. has received research grants from Biogen, Restless Legs Syndrome Foundation, Parkinson's Study Group, Dystonia Coalition (NIH), Cerevel, Cerevance, Ask Bio, and Harmony. He has received honoraria for speaker bureaus from TEVA, Neurocrine, ACADIA, Abbvie, Supernus, and Merz. He has received consulting fees from Merz, Jazz, Neurocrine, Emalex, Supernus, Amneal, Abbvie, Encora, Ipsen, Centessa, and Revance. He receives royalties from the books Movement Disorders in Psychiatry and UpToDate.

## Data Availability

The data that support the findings of this study are available from the corresponding author upon reasonable request.
